# Accelerated brain aging in alcohol users: Insights from machine learning

**DOI:** 10.1111/acer.70049

**Published:** 2025-04-21

**Authors:** Zhenhao Shi, Corinde E. Wiers

**Affiliations:** Department of Psychiatry, University of Pennsylvania Perelman School of Medicine, Philadelphia, Pennsylvania, USA

Alcohol use is highly prevalent and has been associated with a range of health consequences, including disability and mortality. Chronic alcohol consumption can lead to alcohol use disorder (AUD) and cause structural, metabolic, and functional alterations in the brain, resulting in cognitive and behavioral impairments and social problems (Tomasi et al., 2019, 2021; Wang et al., 2024). The relationship between alcohol consumption and brain health is a topic of growing concern, particularly as emerging evidence suggests that relatively moderate drinking, for example, only two units a day, impacts brain volume (Daviet et al., 2022).

Battista et al. (2025) conducted a well-designed cross-sectional study, employing sophisticated neuroimaging techniques and behavioral testing to investigate the role of brain aging in mediating the link between alcohol consumption and cognition. The study by Battista et al. is the first (as noted by the authors) to show that a machine learning-predicted measure of brain aging was associated with a task-based measure of behavioral inflexibility to support highlighting the method's novel features and advantages. Specifically, the authors leveraged an established structural magnetic resonance imaging (MRI)-based machine learning model to explore relationships between brain age, alcohol use severity, and behavioral inflexibility in young, healthy adults (*n* = 58, ages 22–40). They showed that individuals with more hazardous alcohol use, assessed via the self-report Alcohol Use Disorder Identification Test (AUDIT), exhibited significantly more advanced brain aging (i.e., a mean of 1.7years) than those with less severe alcohol use, while correcting for chronological age. The AUDIT scores in the sample were relatively low: the mean AUDIT total score was 4.3, with the total AUDIT threshold of 0–7 indicating low risk, and only *n* = 13 reported an AUDIT score greater than or equal to eight (8), indicating hazardous use. Additionally, they found that high AUDIT scores and accelerated brain aging were both related to poorer behavioral flexibility, as indexed by more errors made on the Hidden Association Between Images Task (HABIT). There was a significant mediation effect, such that individual differences in accelerated brain aging mediated the relationship between AUDIT scores and HABIT task performance. Therefore, aging-related structural brain changes may underlie the harmful impact of alcohol use on cognitive flexibility, a critical aspect of executive function and decision making. These findings contribute to a growing body of literature suggesting that hazardous alcohol consumption may have more pervasive effects on the brain than previously thought, even in young adulthood.

A strength of this study is its use of cutting-edge neuroimaging techniques and machine learning algorithms, which allow for a reliable estimation of brain aging. Brain age estimation has become an increasingly popular approach in neuroscience research as it provides a holistic measure of an individual's brain health, considering various factors beyond chronological age (Kumari & Sundarrajan, 2024; Yang et al., 2024). Most MRI studies of addiction have taken a univariate approach that treats each brain area individually, for example, a voxel or an anatomical region of interest. Although this approach allows for the testing of area-specific effects, it does not allow for testing the integration of effects that jointly produce a clinical or behavioral manifestation (Rashid & Calhoun, 2020). It can also suffer from low statistical power due to excessive corrections needed to account for the number of voxel-wise or region-wise tests (Rashid & Calhoun, 2020). The multivariate approach, as exemplified in Battista et al. (2025), addresses these limitations by characterizing the spatial patterns of structures and functions across multiple brain areas. It is more sensitive in detecting subtle inter-subject variability than the traditional univariate approach and avoids problems such as multiple comparison correction, allowing a large number of brain regions to be examined simultaneously (Rashid & Calhoun, 2020).

Additionally, Battista et al.'s (2025) use of a preestablished multivariate model of brain aging circumvented the need for extensive training and testing samples—a significant advantage for clinical addiction MRI studies, where large sample sizes are often unattainable. The model, originally developed via machine learning by Cole et al. (2019) (https://github.com/james-cole/brainageR), was based on a brain volumetric dataset assessed by structural MRI in adults aged 18–92 years. Similar brain aging models exist, some of which were similarly developed using structural MRI data, while others were based on functional or diffusion MRI data (Kumari & Sundarrajan, 2024). Applying such models to smaller clinical samples mirrors the use of polygenic risk scores derived from large genome-wide association studies, which provide biologically informed metrics—in this case, acceleration of brain aging. Given the established association between gray matter volume reduction and cognitive decline, this methodology offers a meaningful and interpretable measure of neurobiological aging that can be linked to cognitive outcomes. Indeed, Battista et al. (2025) showed that more advanced brain aging accounted for the impact of alcohol use on behavioral inflexibility, confirming the model's external validity.

The utility of this approach extends beyond structural MRI. It holds potential for application across various neuroimaging modalities, including assessments of white matter integrity, task-induced blood oxygen level-dependent (BOLD) response, and structural/functional connectivity (Kumari & Sundarrajan, 2024). Such applications are particularly relevant to substance use research, as alcohol and other drugs impact multiple neurobiological and neuropsychological processes (Kwako et al., 2018). Integrating brain aging models with peripheral markers of aging, such as cardiovascular and immunological indicators or proteomic profiles (Elliott et al., 2021), could offer further insights into biological aging in substance use populations.

Findings from Battista et al. (2025) suggest that brain aging may serve as an intermediate or mechanistic outcome measure for evaluating treatment efficacy and recovery following abstinence. As machine learning models continue to be validated on both research-quality and clinical-quality MRI data (Wood et al., 2022) as well as the more feasible electroencephalogram (EEG) data (Yook et al., 2021), incorporating brain aging metrics into routine assessments could offer objective measures of alcohol-induced brain aging. The findings from Battista et al. (2025) have important implications for public health. They underscore the importance of considering the long-term effects of alcohol consumption on brain health, even in individuals who are not yet in middle or older adulthood. While moderate alcohol consumption may be widely accepted in many societies, the evidence emerging from studies like this suggests that even in young adulthood, alcohol use can have measurable effects on brain structure and cognitive function. This could inform public health campaigns and interventions aimed at reducing hazardous alcohol use among young adults, particularly those in high-risk environments.

While the study sample consisted of healthy young adults, the lack of a more diverse participant pool limits the generalizability of the findings. For example, alcohol consumption was low, and current psychoactive drug use was exclusionary. Furthermore, the accelerated brain aging by a mean of 1.7 years seems moderate, and its impact on daily behavior remains unclear. Future studies would benefit from including a larger sample of individuals with varying levels of alcohol consumption, including those with an AUD. Lastly, the brain-age model used in the study relied solely on structural MRI data. Given the multi-level impact of alcohol use on the brain, future studies that combine structural MRI with other imaging modalities (e.g., functional connectivity [Wang et al., 2024]) will likely offer a more comprehensive understanding of alcohol-induced brain aging.
